# Bis[(1-methyl-1*H*-tetra­zol-5-yl)sulfan­yl]methane

**DOI:** 10.1107/S1600536811011007

**Published:** 2011-03-31

**Authors:** Wei Wei, Zheng-qiang Xia, San-ping Chen, Sheng-li Gao

**Affiliations:** a College of Chemistry and Materials Science, Northwest University, Xi’an 710069, Shaanxi, People’s Republic of China

## Abstract

The mol­ecule of the title compound, C_5_H_8_N_8_S_2_, lies on a twofold rotation axis that relates on 1-methyl­tetra­zolyl group to the other; the five-membered rings are twisted by 53.1 (1)°.

## Related literature

For the synthesis and pharmacological activity of compounds containing tetra­zole groups, see: Semenov (2002 ▶); Upadhayaya *et al.* (2004 ▶). For a related structure, see: Bronisz (2002 ▶).
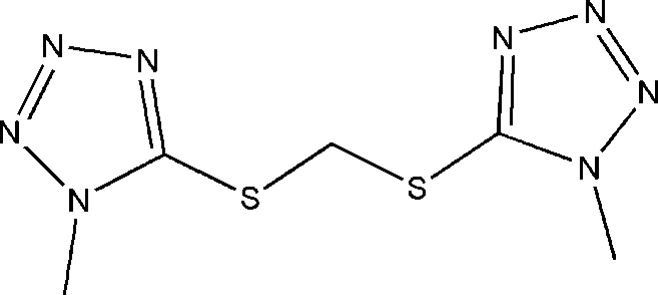


## Experimental

### 

#### Crystal data

C_5_H_8_N_8_S_2_
*M* *_r_* = 244.31
Orthorhombic, 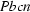

*a* = 6.415 (3) Å
*b* = 7.314 (3) Å
*c* = 22.204 (8) Å
*V* = 1041.9 (7) Å^3^
*Z* = 4
Mo *K*α radiation
μ = 0.49 mm^−1^
*T* = 296 K
0.15 × 0.12 × 0.08 mm

#### Data collection

CBruker SMART area-detector diffractometer
Absorption correction: multi-scan (*SADABS*; Bruker, 2002 ▶) *T*_min_ = 0.930, *T*_max_ = 0.962
4692 measured reflections
936 independent reflections
482 reflections with *I* > 2σ(*I*)
*R*_int_ = 0.118

#### Refinement

*R*[*F* ^2^ > 2σ(*F* ^2^)] = 0.063
*wR*(*F* ^2^) = 0.108
*S* = 1.21
936 reflections
70 parameters
H-atom parameters constrained
Δρ_max_ = 0.34 e Å^−3^
Δρ_min_ = −0.38 e Å^−3^

### 

Data collection: *SMART* (Bruker, 2002 ▶); cell refinement: *SAINT* (Bruker, 2002 ▶); data reduction: *SAINT*; program(s) used to solve structure: *SHELXS97* (Sheldrick, 2008 ▶); program(s) used to refine structure: *SHELXL97* (Sheldrick, 2008 ▶); molecular graphics: *SHELXTL* (Sheldrick, 2008 ▶); software used to prepare material for publication: *SHELXL97*.

## Supplementary Material

Crystal structure: contains datablocks global, I. DOI: 10.1107/S1600536811011007/ng5140sup1.cif

Structure factors: contains datablocks I. DOI: 10.1107/S1600536811011007/ng5140Isup2.hkl

Additional supplementary materials:  crystallographic information; 3D view; checkCIF report

## Figures and Tables

**Fig. 1.**
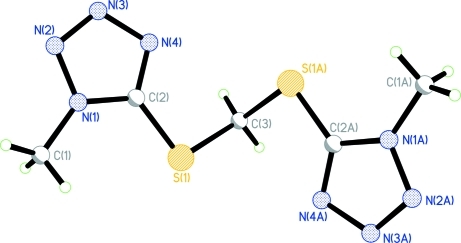
A view of the molecular structure of title compound.

**Fig. 2.**
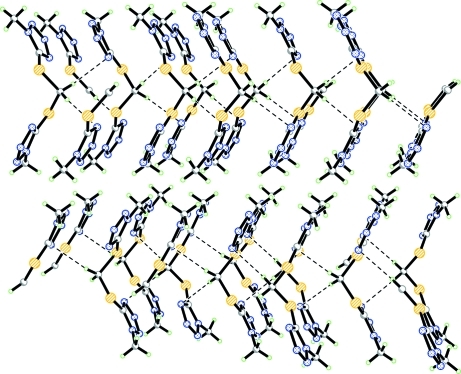
The crystal packing of the title compound.

## supplementary crystallographic information

### Experimental

Sodium hydroxide (1.7 g, 0.043 mol) was added to 5-mercapto-1-methyltetrazole (5 g, 0.043 mol) in dry dimethylsulfoxide (35 ml). The reaction mixture was stirred at 363 K for 1 h. Dichloromethane (3.1 ml, 0.0215 mol) was then added to the solution dropwise with the formation of a grey suspension. The suspension was stirred for 4 h, cooled to room temperature and filtered. The solvent was removed completely under reduced pressure. The residue was recrystallized from ethanol to give a white crystalline product (2.94 g; m.p. 353 - 354 K). Single crystals of the title compound suitable for X-ray diffraction analysis were isolated after a week from a solution in acetone.

### Refinement

All H atoms were positioned geometrically (C—H = 0.96 Å for aromatic CH_3_ and 0.97 Å for CH_2_ groups, respectively) and constrained to ride on their parent atoms with *U*_iso_(H) values set to be -1.5 of the carrier atom.

### Crystal data

**Table:**

C_5_H_8_N_8_S_2_	*F*(000) = 504
*M**_r_* = 244.31	*D*_x_ = 1.558 Mg m^−^^3^*D*_m_ = 1.558 Mg m^−^^3^*D*_m_ measured by not measured
Orthorhombic, *P**b**c**n*	Mo *K*α radiation, λ = 0.71073 Å
Hall symbol: -P 2n 2ab	Cell parameters from 214 reflections
*a* = 6.415 (3) Å	θ = 2.5–18.9°
*b* = 7.314 (3) Å	µ = 0.49 mm^−^^1^
*c* = 22.204 (8) Å	*T* = 296 K
*V* = 1041.9 (7) Å^3^	Flake-like, colourless
*Z* = 4	0.15 × 0.12 × 0.08 mm

### Data collection

**Table:**

CBruker SMART area-detector diffractometer	936 independent reflections
Radiation source: fine-focus sealed tube	482 reflections with *I* > 2σ(*I*)
graphite	*R*_int_ = 0.118
φ and ω scans	θ_max_ = 25.1°, θ_min_ = 1.8°
Absorption correction: multi-scan (*SADABS*; Bruker, 2002)	*h* = −7→7
*T*_min_ = 0.930, *T*_max_ = 0.962	*k* = −8→4
4692 measured reflections	*l* = −25→26

### Refinement

**Table:**

Refinement on *F*^2^	Primary atom site location: structure-invariant direct methods
Least-squares matrix: full	Secondary atom site location: difference Fourier map
*R*[*F*^2^ > 2σ(*F*^2^)] = 0.063	Hydrogen site location: inferred from neighbouring sites
*wR*(*F*^2^) = 0.108	H-atom parameters constrained
*S* = 1.21	*w* = 1/[σ^2^(*F*_o_^2^) + (0.*P*)^2^ + 0.7202*P*] where *P* = (*F*_o_^2^ + 2*F*_c_^2^)/3
936 reflections	(Δ/σ)_max_ < 0.001
70 parameters	Δρ_max_ = 0.34 e Å^−^^3^
0 restraints	Δρ_min_ = −0.38 e Å^−^^3^

### Special details

**Table:**

Geometry. All e.s.d.'s (except the e.s.d. in the dihedral angle between two l.s. planes) are estimated using the full covariance matrix. The cell e.s.d.'s are taken into account individually in the estimation of e.s.d.'s in distances, angles and torsion angles; correlations between e.s.d.'s in cell parameters are only used when they are defined by crystal symmetry. An approximate (isotropic) treatment of cell e.s.d.'s is used for estimating e.s.d.'s involving l.s. planes.
Refinement. Refinement of *F*^2^ against ALL reflections. The weighted *R*-factor *wR* and goodness of fit *S* are based on *F*^2^, conventional *R*-factors *R* are based on *F*, with *F* set to zero for negative *F*^2^. The threshold expression of *F*^2^ > σ(*F*^2^) is used only for calculating *R*-factors(gt) *etc*. and is not relevant to the choice of reflections for refinement. *R*-factors based on *F*^2^ are statistically about twice as large as those based on *F*, and *R*- factors based on ALL data will be even larger.

### Fractional atomic coordinates and isotropic or equivalent isotropic displacement parameters (Å^2^)

**Table:**

	*x*	*y*	*z*	*U*_iso_*/*U*_eq_	Occ. (<1)
S1	1.1330 (2)	1.03366 (18)	0.19301 (6)	0.0521 (4)	
N3	0.6134 (7)	0.9428 (6)	0.1121 (2)	0.0590 (13)	
C2	0.9188 (8)	0.9782 (6)	0.1491 (2)	0.0391 (13)	
N4	0.7207 (7)	1.0077 (6)	0.16107 (17)	0.0498 (12)	
N1	0.9358 (7)	0.8966 (6)	0.09509 (17)	0.0463 (11)	
C1	1.1179 (8)	0.8355 (7)	0.0621 (2)	0.0617 (16)	
H1A	1.1692	0.9336	0.0375	0.093*	
H1B	1.0808	0.7338	0.0369	0.093*	
H1C	1.2242	0.7985	0.0899	0.093*	
N2	0.7401 (8)	0.8743 (6)	0.07305 (18)	0.0561 (13)	
C3	1.0000	1.1654 (9)	0.2500	0.050 (2)	
H3A	1.1011	1.2439	0.2697	0.074*	0.50
H3B	0.8989	1.2439	0.2303	0.074*	0.50

### Atomic displacement parameters (Å^2^)

**Table:**

	*U*^11^	*U*^22^	*U*^33^	*U*^12^	*U*^13^	*U*^23^
S1	0.0481 (9)	0.0665 (10)	0.0418 (8)	0.0008 (8)	−0.0041 (7)	−0.0064 (7)
N3	0.049 (3)	0.061 (3)	0.068 (3)	−0.006 (3)	−0.010 (3)	0.001 (3)
C2	0.050 (4)	0.034 (3)	0.033 (3)	−0.004 (3)	−0.002 (2)	0.004 (2)
N4	0.041 (3)	0.061 (3)	0.047 (3)	0.002 (2)	0.004 (2)	0.004 (2)
N1	0.051 (3)	0.053 (3)	0.035 (2)	−0.004 (2)	−0.003 (2)	−0.003 (2)
C1	0.062 (4)	0.075 (4)	0.049 (3)	0.001 (3)	0.004 (3)	−0.012 (3)
N2	0.051 (3)	0.068 (3)	0.050 (3)	−0.004 (3)	−0.008 (3)	0.000 (2)
C3	0.061 (6)	0.054 (5)	0.034 (4)	0.000	−0.012 (4)	0.000

### Geometric parameters (Å, °)

**Table:**

S1—C2	1.734 (5)	N1—C1	1.450 (6)
S1—C3	1.805 (4)	C1—H1A	0.9600
N3—N2	1.289 (5)	C1—H1B	0.9600
N3—N4	1.372 (5)	C1—H1C	0.9600
C2—N4	1.316 (6)	C3—S1^i^	1.805 (4)
C2—N1	1.343 (5)	C3—H3A	0.9700
N1—N2	1.357 (5)	C3—H3B	0.9700
			
C2—S1—C3	98.31 (19)	H1A—C1—H1B	109.5
N2—N3—N4	110.6 (4)	N1—C1—H1C	109.5
N4—C2—N1	109.4 (4)	H1A—C1—H1C	109.5
N4—C2—S1	127.8 (4)	H1B—C1—H1C	109.5
N1—C2—S1	122.8 (4)	N3—N2—N1	107.1 (4)
C2—N4—N3	105.5 (4)	S1^i^—C3—S1	115.5 (4)
C2—N1—N2	107.5 (4)	S1^i^—C3—H3A	108.4
C2—N1—C1	130.8 (5)	S1—C3—H3A	108.4
N2—N1—C1	121.7 (4)	S1^i^—C3—H3B	108.4
N1—C1—H1A	109.5	S1—C3—H3B	108.4
N1—C1—H1B	109.5	H3A—C3—H3B	107.5

Symmetry codes: (i) −*x*+2, *y*, −*z*+1/2.
